# A Whole-Transcriptomic Analysis of Canine Oral Melanoma: A Chance to Disclose the Radiotherapy Effect and Outcome-Associated Gene Signature

**DOI:** 10.3390/genes15081065

**Published:** 2024-08-13

**Authors:** Greta Mucignat, Ludovica Montanucci, Ramy Elgendy, Mery Giantin, Paola Laganga, Marianna Pauletto, Franco Mutinelli, Marta Vascellari, Vito Ferdinando Leone, Mauro Dacasto, Anna Granato

**Affiliations:** 1 Department of Comparative Biomedicine and Food Science, University of Padua, Agripolis Legnaro, 35020 Padua, Italy; greta.mucignat@phd.unipd.it (G.M.); mery.giantin@unipd.it (M.G.); marianna.pauletto@unipd.it (M.P.); 2 McGovern Medical School and Center for Neurogenomics, UTHealth, University of Texas Houston, Houston, TX 77030, USA; ludovica.montanucci@uth.tmc.edu; 3 Discovery Sciences, Centre for Genomics Research, AstraZeneca, 411 10 Gothenburg, Sweden; ramy.elgendy@astrazeneca.com; 4 Anicura—Centro Oncologico Veterinario, Sasso Marconi, 40037 Bologna, Italy; paola.laganga@anicura.it (P.L.); vito.leone@anicura.it (V.F.L.); 5 Veterinary and Public Health Institute, Legnaro, 35020 Padua, Italy; fmutinelli@izsvenezie.it (F.M.); mvascellari@izsvenezie.it (M.V.)

**Keywords:** melanoma, radiotherapy, RNA-seq, dog, temozolomide

## Abstract

Oral melanoma (OM) is the most common malignant oral tumour among dogs and shares similarities with human mucosal melanoma (HMM), validating the role of canine species as an immunocompetent model for cancer research. In both humans and dogs, the prognosis is poor and radiotherapy (RT) represents a cornerstone in the management of this tumour, either as an adjuvant or a palliative treatment. In this study, by means of RNA-seq, the effect of RT weekly fractionated in 9 Gray (Gy), up to a total dose of 36 Gy (4 weeks), was evaluated in eight dogs affected by OM. Furthermore, possible transcriptomic differences in blood and biopsies that might be associated with a longer overall survival (OS) were investigated. The immune response, glycosylation, cell adhesion, and cell cycle were the most affected pathways by RT, while tumour microenvironment (TME) composition and canonical and non-canonical WNT pathways appeared to be modulated in association with OS. Taking these results as a whole, this study improved our understanding of the local and systemic effect of RT, reinforcing the pivotal role of anti-tumour immunity in the control of canine oral melanoma (COM).

## 1. Introduction

Malignant melanoma is a relatively common cancer of canines, with a high local invasiveness and metastatic propensity, accounting for about 100,000 diagnoses/year in the USA. It most frequently occurs in the oral cavity (COM), hairy skin (cutaneous melanoma), nailbed epithelium, and footpad (subungual and acral melanoma), as well as in ocular tissue (uveal melanoma). The mean age of canines at the diagnosis of malignant melanoma is 11.6 years. Certain canine breeds are more likely to develop melanoma, e.g., Poodles, Beauce Shepherds, Rottweilers, Schnauzers, Scottish Terriers, and Labrador Retrievers; furthermore, black-coated breeds are usually over-represented when compared to white-coated ones. The presence of a protective hair coat suggests that UV does not play a significant role in melanocytic melanoma [1,2,3,4].

As said, COM is the most common oral malignancy in dogs; the most frequent localization is the gingiva, but it can be found in the lip, tongue, and palate. It can be heavily pigmented or amelanotic, ulcerated, and necrotic. Overall, it is an aggressive malignant tumour, locally invasive and highly metastatic, with regional lymph nodes and lungs as preferential metastatic sites [5]. The prognosis is poor, with a median survival time of roughly 65 days in the absence of therapy [6]. As to therapy, the primary conventional treatment is a wide-margin surgery resection, combined (adjuvant) or not (palliative) with radiation therapy (RT), to locally control the tumour. However, such a therapeutic approach is much less successful for metastatic disease [5,6,7]. The use of conventional anticancer drugs is still controversial. For example, platinum compounds and melphalan, administered alone or as adjuvant chemotherapeutics following surgery or RT, show low response rates and did not improve survival times [6,8]. Similarly, unclear and underwhelming results are reported in humans, too [6]. Temozolomide (TEM) deserves a separate discussion. This orally available DNA alkylating prodrug is believed to be a radiosensitizer, and it represents the gold standard for the treatment of human glioblastoma and metastatic melanoma [9,10,11,12]. In canines, TEM shows some anti-tumour activity against melanoma and glioma [13,14]. As classical clinical management approaches are challenging, various immunotherapy strategies such as vaccination (Oncept™, a bacterial plasmid DNA vaccine against human tyrosinase) and monoclonal antibodies (e.g., targeting programmed cell death-1, PD-1) have been used in the therapy of COM. However, controversial or not yet definitive results have been observed. Hence, additional research on the feasibility of different immunotherapy approaches in COM is needed [6,15,16].

Rodent (murine) models classically represent the gold standard for preclinical research in cancer biology and therapeutics; however, they possess some relevant shortcomings and limitations [2,3,8,17,18,19]. Therefore, the need for additional animal models has become of increasing interest within the scientific community. To address this issue, the term ‘comparative oncology’, i.e., the study of naturally occurring cancers in animals as models for human disease, was coined and such a concept represents one of the clearest examples of the “One Health” approach to diseases [8,20,21]. After thirty years, it is a well-established concept that pets, and particularly canines, represent a unique spontaneous animal model for human cancer research [8,17,18,19,22]. COM is among the most common canine malignancies, and today it is considered a clinically faithful animal model for HMM [1,2,3,4,8,18,19,23,24]. At present, there is still a lack of understanding of HMM at the molecular level [25], and understanding the molecular landscapes of cancer is of paramount importance to facilitate early diagnoses and prognoses, develop and optimize effective therapeutic strategies, and improve the patient outcome [19,25,26,27]. As a consequence, obtaining additional information about COM molecular biology and therapy is currently needed and encouraged [8,19,22,28].

In comparative oncology, genomics underwent rapid growth in the past twenty years, particularly with the completion of the first drafts of human and canine genomes in 2003 and 2005, respectively [21,29,30]. Despite the size difference (the canine genome is ~20% smaller than the human one), human and canine genomes are highly homologous (~85%); furthermore (and interestingly), a higher homology exists among the human top 100 genes most frequently mutated in cancer [31]. At present, more complete assemblies of human and canine genomes have been released by the human Genome Reference Consortium and the International Consortium of Canine Genome Sequencing (Dog10K) [32,33].

Radiation therapy is one of the most common approaches to cancer treatment. Either alone or in combination with other therapeutic options (e.g., surgery, anticancer drugs), RT is used in at least two-thirds of cancer treatment regimens, and approximately half of all cancer patients worldwide have undergone RT [34,35,36]. Furthermore, RT is an important curative option to control uncomplicated loco-regional or unresectable/locally advanced tumours, including HMM and COM, too [13,37,38,39]. The RT dogma “One-Size-Fits-All” assumes that every patient has the same opportunity to benefit from RT [35]. However, with such an approach, RT effectiveness is limited by the maximum dose tolerated by adjacent normal tissues, which may result in radiotoxicity [34,36]. Nowadays, the major goal of RT is to maximize cancer cell killing by using dosage regimens that adjacent healthy tissues can tolerate, with minor radiation injury [36]. To achieve this goal, radiation sensitizers (i.e., drugs responsive to ionizing radiation and enhancing RT effectiveness such as TEM and gemcitabine) have been used in the RT–drug combined treatment of human and canine glioblastoma and metastatic melanoma [9,10,11,13,14,40]. All this allowed RT to evolve from the aforementioned dogma to a more dynamic and patient-tailored therapeutic approach [34,36,41,42].

The sequencing of human and canine genomes paved the way for the era of precision or personalized medicine (PM), whose motto is “the right treatment for the right patient at the right time” [43]. In such a context, next-generation sequencing (NGS) methodologies (i.e., genomics, transcriptomics, epigenomics) are increasingly used in oncology to speed up cancer early diagnoses, discover therapeutic targets, and predict the patient outcome, ultimately improving the PM approach to cancer [44,45,46,47]. Conversely, and surprisingly enough, NGS has not yet similarly affected RT [35]. Thus, the concept of personalized radiation therapy, to guide exclusive RT and/or combination therapy, has emerged and has been encouraged in recent years [44,48,49]. By using NGS datasets, radiation oncologists could identify biomarkers (e.g., gene expression profiles) useful to classify radio-sensitive/resistant tumours and/or tumour-surrounding normal tissues before the treatment [36,44,50]. Gene signatures, useful to identify radiosensitive patients and predict the diagnosis, prognosis, or response to RT, have been developed and validated in some human tumours, e.g., glioblastoma and breast and colorectal cancer cells. To achieve this goal, most studies used candidate gene biomarkers or functional assays of DNA damage repair to predict radiosensitivity. However, few genetic biomarkers are being used to tailor human cancer RT [48,49,50].

To the best of our knowledge, there is still no effective radiosensitive gene signature for COM; only one paper matching whole exome and transcriptome sequencing to identify the possible causes of RT and adjuvant chemotherapy failure has been published so far [51]. Therefore, in this study, we utilized an NGS approach (i.e., RNA-seq) to evaluate the transcriptomic landscape of COM in buccal biopsies before and after RT, and its association with the disease outcome.

In addition, there is an increasing demand for molecular tools to provide clinically valuable information about patient outcomes and successful delivery of PM [52]. In such a context, peripheral blood testing is an alternative approach to invasive needle-based biopsy sampling, especially for tumours that are anatomically difficult to be sampled (such as HMM and COM). Blood sampling is also routinely performed in the clinic and is affordable [52,53,54]. As to COM, to our knowledge, there are no biomarkers in routine clinical use to monitor the canine response to RT; hence, we also investigated the usefulness of a peripheral blood transcriptome as a suitable surrogate tissue for COM.

## 2. Materials and Methods

### 2.1. Animal Recruitment, Clinico-Pathological Features, Therapy, and Sample Acquisition

This study included client-owned dogs affected by COM and presented at the Centro Oncologico Veterinario (Sasso Marconi, Bologna, Italy). For each dog, data collected included sex, age, breed, tumour localization, and size as well as tumour-node metastasis (TNM) staging, according to WHO classification [23].

For all cases, all owners signed written informed consent for the use of the biological samples for research purposes. Approval by an ethics committee was not required, since the research did not influence any diagnostic or therapeutic decision.

During surgery, dogs underwent a surgical biopsy (TruCut or Punch), and the specimen (T_0_) was divided into two aliquots: The first one (~100 mg; T_0_) was placed in Eppendorf tubes filled with 1.8 mL RNAlater™ Stabilizing Solution (Life Technologies, Foster City, CA, USA) and conserved at −20 °C until the analysis. The second aliquot was formalin-fixed (10% buffered formalin) and paraffin-embedded for routine histological and immunohistochemical investigations. A second biopsy (T_1_) was collected at the end of the hypofractionated RT; once placed in an RNA stabilizing solution, it was kept at −20 °C until RNA extraction. Simultaneously, blood samples (in duplicate) were withdrawn in PAXgene tubes (Qiagen, Milan, Italy) as per the manufacturer’s instructions. These were at first stored overnight at room temperature and then conserved at −20 °C until RNA isolation. Tissue and blood sampling procedures were kept consistent for all the samples and were obtained by the same hospital personnel.

The diagnosis of COM was made on hematoxylin and eosin-stained slides by adopting previously proposed criteria, which include the mitotic index (MI), the melanin abundance, and the nuclear atypia grading [55,56,57]. Confirmatory immunohistochemical investigations were made by using antibodies raised against Melan-A (MEL-A) and PNL-2 melanocytic markers as well as Ki-67 (the latter for prognostic purposes) [23,55,56,57]. Only cases diagnosed as COM were taken into consideration.

As to the chosen therapeutic approach, only dogs receiving RT and adjuvant chemotherapy with TEM were included in this study. Dogs were irradiated weekly with fractions of 9 Gray (Gy), up to a total dose of 36 Gy (4 weeks). Two weeks after the completion of hypofractionated RT, dogs received adjuvant chemotherapy with TEM, i.e., 100 mg/m^2^ orally administered once a day and for 5 consecutive days.

To assess the possible relationships between RNA-seq outputs, the treatment protocol, and the patient outcome, the OS, which is a gold standard endpoint in oncology [58], was considered. Specifically, according to the oncologist, a mortality threshold value of 6 months (before/after) was applied to divide the clinical cases into two subgroups. Therefore, all dogs were scrupulously followed up with and the disease outcome was recorded.

### 2.2. RNA Isolation from Biopsies and Blood Samples

RNA from biopsies was isolated using the TRIzol reagent (Life Technologies, Milan, Italy) and subsequently purified using the RNeasy Mini kit (Qiagen, Milan, Italy) as per the manufacturer’s instructions. RNA from blood samples was isolated following the protocol of the PAXgene blood RNA kit (PreAnalytics/Qiagen, Milan, Italy). A DNase treatment was performed on the column for quality assurance before RNA was eluted from the filter. RNA concentration was determined using the NanoDrop ND-1000 UV-Vis spectrophotometer (NanoDrop Technologies Inc., Wilmington, DE, USA), and its quality was measured by the 2100 Bioanalyzer and the RNA 6000 Nano kit (Agilent Technologies, Santa Clara, CA, USA). Only RNA samples with an RNA integrity number (RIN) ≥ 7 were selected for the RNA-seq library preparation. The isolated RNA was stored at −80 °C until further use.

### 2.3. RNA-Seq Library Preparation and Sequencing

Using 1 (blood samples) or 0.5 µg (biopsies) of total RNA as input, mRNA was enriched using the NEB magnetic mRNA isolation kit (New England BioLabs, Ipswich, MA, USA); hence, RNA-seq libraries were prepared using the NEBNext Ultra RNA Library Prep Kit from Illumina (New England BioLabs, Ipswich, MA, USA) as per the manufacturer’s specifications. The prepared libraries were purified with Agencourt Ampure XP beads (Beckman Coulter, Brea, CA, USA). Individual libraries were multiplexed together in a 6-library pool. The pooled libraries were sequenced on 50-cycle runs using an Illumina Hiseq2500 platform (Genomix4Life, Caserta, Italy).

### 2.4. Differential Expression Analysis and Functional Analysis

Raw reads underwent quality control with FastQC software (v.0.11.9; [59]) and low-quality reads and adapters were removed using Trimmomatic (v.0.36; [60]).

Trimmed reads were pseudoaligned to the reference canine transcriptome (ROS_Cfam_1.0, Ensembl release 111) using Kallisto (v.0.48.0; [61]). Transcripts were imported in Rstudio (R version 4.3.0) and collapsed to genes using the tximport package (v.1.30.0; [62]) retrieving the annotations from Ensembl through the R interface biomaRt (v.2.58.0; [63]).

The following steps of the differential gene expression (DGE) analysis were carried out using the edgeR package (v.4.0.3; [64]). The data were organized in four different datasets, the first two comparing T1 vs. T0 for each tissue, using a paired design, and the other two taking into consideration the OS of each subject and setting the contrast between the group with long and short survival time (LONG: >6 months, comprising 3 dogs; SHORT: <6 months, comprising 5 dogs). For these last two comparisons, one tissue at a time was considered and only samples belonging to T0 were analyzed. All the datasets were processed with the same approach as reported below. First, genes with very low expression levels were removed (*filterByExpr*), and the remaining ones were normalized using the *calcNormFactors* function according to the trimmed mean of M-values (TMM).

After common and tagwise dispersion estimation (*estimateDisp*), negative binomial generalized linear models were fitted (*glmFit*), and differentially expressed genes (DEGs) were identified using the likelihood ratio-test (*glmLRT*) setting these contrasts: blood T1 vs. T0 (B_T1vsT0), tumour T1 vs. T0 (T_T1vsT0), blood LONG vs. SHORT (B_OS), and tumour LONG vs. SHORT (T_OS). Differentially expressed genes were defined as those with a Benjamini–Hochberg adjusted *p*-value (BHp) < 0.05 and log_2_ fold change (lfc) > 0.58 or <−0.58 from each dataset.

Moreover, the *gseGO* function from the clusterProfiler package (v.4.10.0; [65]) was applied to perform the Gene Set Enrichment Analysis (GSEA). A list of genes produced by the *glmLRT* function was used to create the input file for this computational analysis, pre-ranking all genes according to their BHp using “1-BHp” and “-(1-BHp)” to include the direction of their expression in the analysis (up- or downregulation, respectively).

### 2.5. Spearman’s Correlation

All DEGs found to be modulated in LONG vs. SHORT comparison in biopsies were submitted to a Spearman nonparametric correlation analysis to check for possible correlation between transcriptional expression (log_2_ counts per million, logCPMs, after filtering and TMM normalization) and OS (days).

### 2.6. Statistical Analysis

Except for RNA-seq data, the statistical analysis was made using the GraphPad Prism software (version 9.5.1, San Diego, CA, USA).

## 3. Results

### 3.1. Clinico-Pathological Features

In a period of time between 1 September 2014 and 31 March 2016, a total of nineteen presumed COM samples were collected. Following a routine TNM staging, the assessment of MI, the presence of melanin pigment, the grading of nuclear atypia, and the immunohistochemical positivity for Melan-A, PNL-2, and Ki-67, a cohort of twelve confirmed COM-affected dogs were initially considered for this study. All the recruited dogs received RT and adjuvant chemotherapy. Following the adoption of this more stringent criteria to standardize the caseload, including the use of TEM as adjuvant chemotherapy, the number of eligible COM was reduced to eight, specifically, two mixed breeds, two Golden Retrievers, one miniature Schnauzer, one miniature Spitz, one Labrador, and one Italian Hound. Five were males, three were females, and two spayed subjects were considered, one for each sex; the age ranged between 8 and 15 years, and the weight was between 6 and 38 kg. A detailed description of clinical and morphological/immunophenotypical features is reported in Tables S1 and S2, while examples of a positive/negative presence of melanin pigment as well as of MEL-A-, PNL-2-, and ki-67-positive immunohistochemical reactions are reported in Figures S1 and S2. Furthermore, an illustrative picture showing the clinical effects of RT + TEM is shown in Figure S3.

### 3.2. Differential Expression Analysis and Functional Analysis

For each sample, considering both biopsies and blood, a mean of 35,090,610 raw reads were obtained and more than 24 million reads (24,754,070 reads on average) were pseudoaligned to the reference transcriptome (Tables S3 and S4).

**Figure 1:**
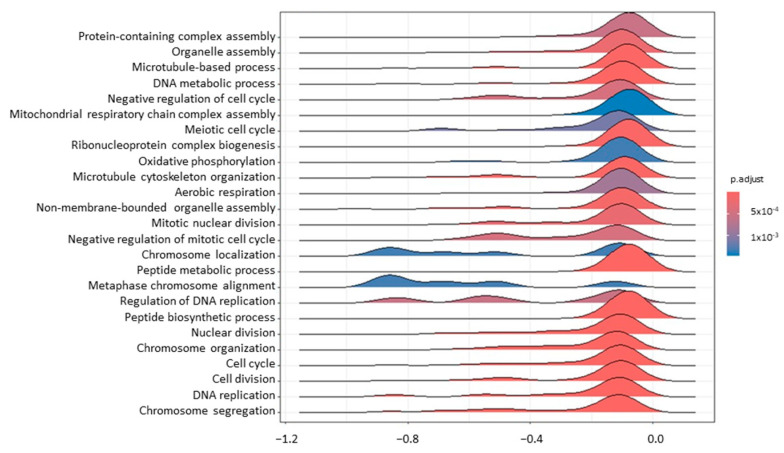
A ridgeplot of the 25 most significant terms as results of GSEA comparing tumour T1 vs. T0. The colour gradient is related to the level of significance, adjusted with the Benjamini–Hochberg method.

Concerning the comparison between T1 and T0 to assess the transcriptional effect of RT, 8 and 109 DEGs were identified in tumour biopsies and blood samples, respectively (Table S5). DEGs for which a reference gene name was available are reported in Table 1 and Table 2. Forty-eight GO terms were found to be enriched for T_T1vsT0, and 14 for B_T1vsT0 (Table S6). As it is possible to appreciate from Figure 1, in tumour samples, the GO terms related to the cell cycle (GO:0007049; GO:0045786), DNA replication (GO:0006260; GO:0006275), and DNA damage response (GO:0006974) were mostly affected by RT. Such modulation is supported by genes found to be differentially expressed, like Cyclin-Dependent Kinase Inhibitor 1A (*CDKN1A*; lfc_T_T1vsT0_ = 1.03), DEAD-Box Helicase 43 (*DDX43*; lfc_T_T1vsT0_ = −6.15), Centromere Protein K (*CENPK*; lfc_T_T1vsT0_ = −1.25), and Metallothionein 1E (*MT1E*; lfc_T_T1vsT0_ = 1.05).

**Figure 2:**
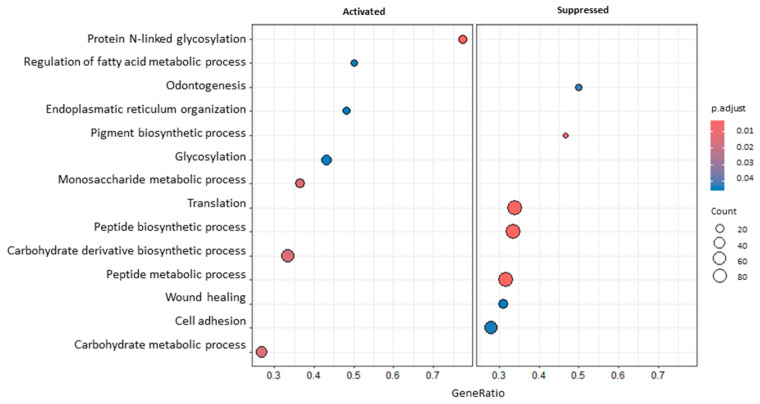
A dotplot of GSEA results comparing blood T1 vs. T0. The dot size represents the number of genes belonging to each pathway. The colour gradient is related to the level of significance, adjusted with the Benjamini–Hochberg method. The box on the left collects activated pathways, while the box on the right collects suppressed ones.

Some of the most interesting GO terms modulated after RT in blood samples (Figure 2) were related to glycosylation (GO:0006487, GO:0070085), translation (GO:0006412), cell adhesion (GO:0007155), and wound healing (GO:0042060). Looking more in detail into the list of DEGs, several transcripts possibly linked to the immune microenvironment and radiosensitivity were noticed, e.g., C-C Motif Chemokine Receptor 7 (*CCR7*; lfc_B_T1vsT0_ = −0.89), the Interleukin 21 Receptor (*IL21R*; lfc_B_T1vsT0_ = −0.92), the Thymocyte Selection-Associated High-Mobility Group Box (*TOX*; lfc_B_T1vsT0_ = −0.81), Layilin (*LAYN*; lfc_B_T1vsT0_ = 1.01), Lymphoid Enhancer Binding Factor 1 (*LEF1*; lfc_B_T1vsT0_ = −0.9), Purinergic Receptor P2Y2 (*P2RY2*; lfc_B_T1vsT0_ = 0.78), MLX Interacting Protein-Like (*MLXIPL*; lfc_B_T1vsT0_ = 0.99), Suppressor of Cytokine Signaling 3 (*SOCS3*; lfc_B_T1vsT0_ = −0.72), and KLF Transcription Factor 10 (*KLF10*; lfc_B_T1vsT0_ = 0.79). In support of GSEA results, it is important to also mention some genes involved in cell adhesion, e.g., Integrin Subunit β 5 (*ITGB5*; lfc_B_T1vsT0_ = −0.59) and Junctional Adhesion Molecule 3 (*JAM3*; lfc_B_T1vsT0_ = −0.81).

In order to discern potential transcriptional features of COM specimens that might be suggestive of a prognosis at baseline, LONG vs. SHORT comparisons were considered. In this respect, T0 samples were only analyzed to avoid any biases due to the therapeutic intervention. No terms were enriched either in blood or biopsies. Nevertheless, it was possible to identify 18 and 4 DEGs according to the OS in tumour and blood tissue, respectively (Table S5). The list of DEGs for which a reference gene name was available is reported in Table 3 and Table 4. As to tumour samples, interesting upregulated genes were Keratin 76 (*KRT76*; lfc_T_OS_ = 8.59); Integrin Subunit α 8 (*ITGA8*; lfc_T_OS_ = 5.64); Microfibril-Associated Protein 5 (*MFAP5*; lfc_T_OS_ = 4.52); Sushi, von Willebrand Factor Type A, EGF, and Pentraxin Domain Containing 1 (*SVEP1*; lfc_T_OS_ = 3.71); and Sclerostin Domain Containing 1 (*SOSTDC1*; lfc_T_OS_ = 4.66). Among the downregulated ones, worthy of mention is the modulation of Matrix Metallopeptidase 13 (*MMP13*; lfc_T_OS_ = −4.29) and Wnt Family Member 5B (*WNT5B*; lfc_T_OS_ = −2.74). Concerning blood samples, we highlight the upregulation of Transketolase-Like 1 (*TKTL1*; lfc_B_OS_ = 3.04).

### 3.3. Spearman’s Correlation

The Spearman’s test was applied to investigate the possible correlation between the expression of DEGs regulated in T_OS and OS (Table 5). The expression of *MMP13*, *WNT5B*, *SVEP1*, and phospholipase A2 group IVF (*PLA2G4F*) was significantly correlated with the OS. As shown in Figure S4, the first and the last two genes of the abovementioned list appear to be positively and negatively correlated with the OS, respectively.

## 4. Discussion

### 4.1. Transcriptomic Effect of RT on Blood and Tumour Samples

RT represents a cornerstone in the management of COM for local tumour control, through a direct action on cancer cells and immunomodulatory effects. Such an anticancer immune response could be within or outside the radiation field. Nevertheless, this abscopal activity is often incomplete and inefficient without the use of an adjuvant therapy. Canine melanoma as well as osteosarcoma are immunogenic tumours, and could represent priceless models to define possible strategies to amplify the effect of specific anticancer treatments. Just these pieces of evidence pushed us to explore more in-depth how RT could modulate the COM microenvironment and, consequently, increase the patients’ sensitivity to any adjuvant chemotherapies like TEM or to immunotherapies [66,67,68].

Starting from comparing T1 and T0 in biopsies, we could appreciate one of the well-known effects associated with RT. Indeed, radiation causes DNA damage and consequently affects the cell cycle [66]. In the present study, such an effect was confirmed by these specific enriched terms. Moreover, *CDKN1A*, coding for p21 protein, was found to be upregulated. Specifically, this gene is transcriptionally modulated by p53 in response to DNA damage guiding G1 cell cycle arrest [69].

Furthermore, the treatment downregulated *DDX43* and *CENPK*. The former gene is a DEAD-box RNA-helicase family member associated with adverse clinico-pathological characteristics in breast cancer and lung adenocarcinoma [70,71,72]. Likewise, *CENPK* is a kinetochore protein and its overexpression in hepatocellular carcinoma promotes proliferation and in ovarian cancer is associated with a poor prognosis [73,74].

Interestingly, *MT1E* was positively modulated by RT. Metallothioneins are proteins playing a major role in metal ion homeostasis and detoxification. In cancer, they can act as antioxidants, protecting cells from free radicals generated by mutagens, antineoplastic drugs, and radiation [75]. Furthermore, its expression is regulated by methylation in melanoma, being a potential tumour suppressor gene [76].

After exploring the effect of RT on the cell cycle in the tumour tissue, in blood samples, it was possible to investigate other aspects of this therapeutic approach. The enrichment of glycosylation-related terms after RT aligns with what is reported in the literature. Indeed, it has been shown that ionizing radiation causes changes in protein glycosylation; in particular, such a post-translational modification could influence cell adhesion [77,78]. In this case, vasculature and more specifically the endothelium–monocyte interaction could play a pivotal role in the response to RT [78].

It is already known how the TME controls the growth and progression of cancerous tissue; therefore, the impact of RT on vascularization and immune cells could influence the prognosis and the effect of adjuvant treatments. One of the main immune subset populations targeted by RT are CD8+ T cells, and these often contribute to the abovementioned abscopal effect [79]. In line with this, in the present study, several genes possibly involved in CD8+ function and differentiation were modulated by RT, e.g., *P2RY2*, *LAYN*, and *MKXIPL*. Specifically, the first one is a receptor activated by ATP released passively from dead cells and acting as a pro-inflammatory signal. We could speculate that RT, causing the death of cancerous cells, leads to an ATP-driven and purigenic receptor-dependent accumulation of CD11c + CD11b + Ly6Chi tumour-infiltrating leukocytes. These cells appear to be particularly efficient at presenting tumour antigens to CD8+ T cells, according to what was previously reported by Ma et al. [80,81]. In human melanoma, *LAYN* is one of the most enriched genes among the phenotypically exhausted yet clonally expanded tumour-infiltrating lymphocytes. The deletion of this gene causes enhanced tumour growth and the coded protein facilitates the effector capability of cytotoxic T cells, pointing out that the amount and the specific type of CD8+ T cells present in a cancerous tissue contribute to the patient’s immune response to cancer. However, in contrast with our findings, it seems to not be present in peripheral blood cells [82]. In support of these data, and in line with a possible higher infiltration of CD8+ T cells, *MLXIPL* was also upregulated by RT, despite its low constitutive expression level. This gene has a multifaceted role depending on the type of tumour and in prostate cancer, its expression is induced by T cell infiltration [83]. Furthermore, an additional sign of the modulation of CD8+ population function comes from the downregulation of *IL21R*, *LEF1*, *TOX*, and *CCR7* genes [84,85,86].

Other genes modulated by RT in blood samples and worthy to be cited are *SOCS3* and *KLF10*. The first one, found to be inhibited in the present work, is supposed to play a role in radioresistance; indeed, the silencing of this gene affects radioresistance in glioblastoma cell lines [87]. Intriguingly, it is inducible by different interleukins (e.g., IL-21) through the JAK/STAT pathway, on which it acts as a negative feedback regulator [88]. *KLF10*, upregulated by RT, acts as a tumour suppressor gene in several cancers through the TGF-β signalling pathway [89]. Furthermore, like *SOCS3*, it is considered a possible marker of radiosensitivity in pancreatic adenocarcinoma, transcriptionally suppressing the UV radiation resistance-associated gene (*UVRAG*) and modulating apoptosis, DNA repair, and autophagy [90].

Another aspect that emerged from our analysis is the impact on cell adhesion molecules. The *JAM3* and *ITGB5* genes were downregulated by RT and the adhesion-related pathways were enriched, too. In a former study, *ITGB5* was found to be differentially expressed in the NCI-60 cancer cell panel, thus suggesting that adhesion molecules could have a major role in radiosensitivity [91]. The mRNA levels of *JAM3* are higher in metastatic malignant melanomas and its expression is correlated with invasive properties and metastatic potential either in fibrosarcoma or melanoma and also in bladder cancer cell lines [92,93,94,95]. Additionally, it has been demonstrated that the occurrence of lung metastasis of melanoma could be partially mediated by the interaction between jam-b (*JAM2*) and jam-c (*JAM3*) [96]. Finally, *JAM3* is negatively associated with CD8+ cell infiltration in bladder cancer samples [95], while integrins significantly correlate with immune cell infiltration in skin cutaneous melanoma [97], bringing our discussion back to antineoplastic immunity, the leitmotif of these results.

### 4.2. Transcriptomic Differences Associated with Overall Survival Time

To identify potential biomarkers of aggressiveness, which could be related to OS, the transcriptome of subjects with long vs. short OS was compared. Some hints suggest that TME is probably one of the main factors affecting the OS. As a matter of fact, it comprises immune and stromal cells, the extracellular matrix, and blood and lymphatic vessels, and plays a critical role in survival and therapeutic response [98]. Two possible markers of cancer-associated fibroblasts (CAFs), *MFAP5* and *SVEP1*, were upregulated in biopsies, and the expression of the last one was positively correlated with a longer OS. Cancer-associated fibroblasts belong to an abundant and heterogeneous cell population of tumour stroma possessing several pro-tumour and tumour-suppressing functions, according to a specific cell subpopulation. In skin cancers, CAFs express different markers and in advanced melanoma, they could be involved in resistance to immunotherapy and BRAF inhibitors [98,99,100]. In a former study, *MFAP5* and *SVEP1* have been related to a subset of CAFs with a strong extracellular matrix signature in a mouse model of MMTV-PyMT (mouse mammary tumour virus–polyoma middle tumour antigen) [101]. The first gene (*MFAP5*) is a microfibril-associated glycoprotein involved in the deposition of elastic microfibrils. Its dysregulation seems to assume different meanings according to the compartment of the tumour involved (epithelial or stromal part) and the tumour type taken into consideration. Overexpression in the epithelial counterpart of this gene in head and neck squamous cell carcinoma, ovarian, and breast cancer is related to a worse prognosis [102,103,104]. Moreover, in ovarian cancer, there is a stromal upregulation of its coded protein (microfibrillar-associated protein 5), being associated with poor survival [105]. However, contradictory results have been published, too: a significant reduction in *MFAP5*/mfap5 was noticed in the stroma of prostate cancer, invasive colonic cancer, and gallbladder adenocarcinoma [106,107,108]. On the other hand, *SVEP1* is an adhesion molecule. In hepatocellular carcinoma, its downregulation leads to a poor prognosis and early recurrence, correlating negatively with cancer stem cell markers (i.e., CD44, CD133) [109,110]. In line with this evidence, in intrahepatic cholangiocarcinoma the level of *SVEP1* dramatically decreases in tumour tissue if compared with the adjacent one, thus correlating with abnormal neovascularization [111]. In the present study, the expression of this gene was positively correlated with OS (*p* < 0.0004), thus confirming the studies cited above. On the other hand, an upregulation of *SVEP1* in CAFs compared to normal fibroblasts, and its involvement in chemoresistance to gemcitabine and cisplatin, has been recently described in urothelial bladder cancer [112]. Nevertheless, it should be emphasized that the present study considered entire tumour samples including both parenchyma and stroma; thus, it is not possible to attribute these transcriptomic changes specifically to one of the two compartments. To propose a definite conclusion, a more in-depth analysis on a homogeneous cell population should be performed.

Also, other genes associated with metastasis and cancer progression were differentially regulated in tumour tissue, i.e., *MMP13*, *WNT5B*, and *SOSTDC1*. Long OS was negatively correlated with *MMP13* and *WNT5B*, an interstitial collagenase and a WNT ligand, respectively. In line with this, in several human cancers including melanoma, *MMP13* is associated with metastasis and poor survival. Intriguingly, in melanoma cell lines this gene enhances invasiveness and proliferation, surprisingly limiting vasculogenic mimicry through the degradation of vascular endothelial cadherin (VE-cadherin or cadherin-5), probably leading to the release of β-catenin in the cytoplasm and nucleus [113,114,115]. Also, *WNT5B* may decrease VE-cadherin expression, having a pro-metastatic role and causing functional and transcriptional changes in lymphatic endothelial cells in oral squamous cell carcinoma (OSCC) and melanoma [116,117]. In addition, its expression correlates with OS in osteosarcoma, hepatocellular carcinoma, and breast cancer [118,119,120]. An antagonist of the WNT/b-catenin pathway, i.e., *SOSTDC1*, was upregulated. *SOSTDC1* restrains the proliferative ability by promoting the apoptotic rate in acute myeloid leukemia [121]. Its downregulation is related to a poor prognosis in several cancer types, e.g., breast and gastric cancer [122]. Overall, this could let us speculate that the canonical and non-canonical WNT pathways could be involved in determining the OS in COM.

In the biopsies belonging to the group with longer OS, it is interesting to highlight the upregulation of genes like *KRT76* and *ITGA8*. The first one, encoding for a structural protein and in agreement with other authors’ opinion, is the most significantly downregulated gene in human OSCC, strongly correlating with a poor prognosis [123]; moreover, it possesses an immunomodulatory role, too [124]. On the other hand, according to the published literature, the level of expression of *ITGA8* is considered to be closely related to metastasis in skin cutaneous melanoma and also correlated with CD8+ infiltration [97]. This gene belongs to the integrin family of transmembrane cell surface receptors, and it is retained to be involved in the process of carcinogenesis in different and sometimes opposite ways, according also to the type of cancer. In ER-positive breast and bladder cancers, it seems to be regulated by methylation, and its consequent lower expression is associated with adverse OS [125].

Finally, taking into consideration the results related to OS in blood samples, *TKTL1* was induced in subjects with longer OS. This gene codes for a homodimeric transketolase, generally overexpressed in cancer cells for the acquisition of a glycolytic phenotype (the Warburg effect); in melanoma, it was proved to be regulated by methylation in vitro [126]. However, apparently in contrast with what emerged in our study, in which a higher expression of *TKTL1* seemed to be associated with longer OS, this gene could be considered a marker of a poor prognosis in different types of cancers [127].

## 5. Conclusions

Drawing some of the main lines of the discussion, it was possible to speculate a possible role of TME in COM progression and response to RT, starting from the modulation of the immune response after the ionizing treatment and up to the role of different TME populations in OS. Noteworthily, the glycosylation, cell adhesion, and cell cycle also appeared to be involved in the response to RT, while the canonical and non-canonical WNT pathways could be essential factors in determining OS. In any case, it is fundamental to highlight that the hypotheses of this study are based on a limited number of subjects, even if characterized by a homogeneous therapeutic approach. Evaluations on a wider dataset of samples should be performed to confirm these results and other types of analyses could be considered to corroborate them. For example, the involvement of different immune populations and the expression peculiarities of normal/cancerous melanocytes and the stroma could be examined, taking into consideration the mutational burden of each tumour.

## Figures and Tables

**Table 1:** Genes modulated by radiotherapy in biopsies (T) (T1 vs. T0). Only the DEGs for which a reference gene name was available are listed. Ensembl gene ID, gene name, gene description, log2 fold change (lfc), log2 counts per million (logCPMs), and Benjamini–Hochberg adjusted *p*-value (BHp) are reported for all the DEGs.

Ensembl Gene ID	Gene Name	Gene Description	lfc	logCPM	BHp
ENSCAFG00845016275	*GNGT1*	G protein subunit γ transducin 1	−1.88	5.07	0.01
ENSCAFG00845030209	*SLCO2A1*	solute carrier organic anion transporter family member 2A1	2.79	3.23	0.01
ENSCAFG00845011968	*DDX43*	DEAD-box helicase 43	−6.15	0.54	0.03
ENSCAFG00845009606 ^1^	(*CDKN1A*) ^2^		1.03	5.43	0.03
ENSCAFG00845027527 ^1^	(*LOC612587*) ^2^		4.63	1.32	0.03
ENSCAFG00845009602	*CENPK*	centromere protein K	−1.25	3.51	0.03
ENSCAFG00845003828	*MT1E*	metallothionein 1E	1.05	6.40	0.03
ENSCAFG00845015023 ^1^	(*C20H3orf14*) ^2^		−2.13	0.85	0.03

^1^ Novel gene; ^2^ UniProtKB Gene Name Symbol.

**Table 2:** Genes modulated by radiotherapy in blood samples (B) (T1 vs. T0). Only the DEGs for which a reference gene name was available are listed. Ensembl gene ID, gene name, gene description, log2 fold change (lfc), log2 counts per million (logCPMs), and Benjamini–Hochberg adjusted *p*-value (BHp) are reported.

Ensembl Gene ID	Gene Name	Gene Description	lfc	logCPM	BHp
ENSCAFG00845008674	*ADAMTS2*	ADAM metallopeptidase with thrombospondin type 1 motif 2	3.09	1.78	0.001
ENSCAFG00845007491	*PROK2*	prokineticin 2	−1.77	6.79	0.023
ENSCAFG00845016746	*XKRX*	XK related X-linked	−1.35	−0.59	0.003
ENSCAFG00845005455	*SLC28A3*	solute carrier family 28 member 3	−1.26	4.00	0.002
ENSCAFG00845026869	*AMIGO2*	adhesion molecule with Ig-like domain 2	−1.24	−0.90	0.038
ENSCAFG00845001908	*KANK1*	KN motif and ankyrin repeat domains 1	−1.21	−0.27	0.001
ENSCAFG00845008460	*TNFAIP6*	TNF α-induced protein 6	−1.17	1.25	0.008
ENSCAFG00845008349	*CD72*	CD72 molecule	−1.14	0.70	0.027
ENSCAFG00845016972	*MYBPC2*	myosin binding protein C2	−1.13	−0.58	0.012
ENSCAFG00845015333	*COL4A4*	collagen type IV α 4 chain	−1.10	−0.50	0.029
ENSCAFG00845028251	*MYO18B*	myosin XVIIIB	1.08	0.85	0.023
ENSCAFG00845015341	*DLGAP3*	DLG-associated protein 3	−1.06	1.19	0.000
ENSCAFG00845008907	*CRIP3*	cysteine-rich protein 3	−1.05	−0.22	0.018
ENSCAFG00845028917 ^1^	(*LOC119864113*) ^2^		−1.02	0.53	0.044
ENSCAFG00845002458	*LAYN*	layilin	1.02	1.32	0.030
ENSCAFG00845028881 ^1^	(*LOC119864112*) ^2^		−1.01	7.64	0.000
ENSCAFG00845030285	*STAB1*	stabilin 1	1.00	2.46	0.003
ENSCAFG00845003394	*MLXIPL*	MLX interacting protein-like	0.99	−0.70	0.033
ENSCAFG00845014560	*AASS*	aminoadipate–semialdehyde synthase	−0.98	3.14	0.045
ENSCAFG00845030320	*SLAMF1*	signalling lymphocytic activation molecule family member 1	−0.98	2.49	0.008
ENSCAFG00845001649	*NIM1K*	NIM1 serine/threonine protein kinase	−0.94	0.87	0.007
ENSCAFG00845015409	*CABLES1*	Cdk5 and Abl enzyme substrate 1	−0.93	0.42	0.012
ENSCAFG00845004274	*IL21R*	interleukin 21 receptor	−0.92	3.23	0.042
ENSCAFG00845006782	*CRISP2*	cysteine-rich secretory protein 3	−0.91	3.11	0.006
ENSCAFG00845015621	*PSD3*	Pleckstrin and Sec7 domain containing 3	−0.90	3.88	0.000
ENSCAFG00845029249	*LEF1*	lymphoid enhancer binding factor 1	−0.90	5.77	0.025
ENSCAFG00845014724	*GCH1*	GTP cyclohydrolase 1	−0.90	2.19	0.038
ENSCAFG00845007930	*CCR7*	C-C motif chemokine receptor 7	−0.89	4.80	0.005
ENSCAFG00845000734	*SLC23A1*	marginal zone B and B1 cell-specific protein	0.86	4.00	0.000
ENSCAFG00845008805	*SCML4*	Scm polycomb group protein-like 4	−0.86	2.37	0.000
ENSCAFG00845028411	*TFDP2*	transcription factor Dp−2	−0.85	6.51	0.004
ENSCAFG00845028315	*B3GALNT1*	β-1,3-N-acetylgalactosaminyltransferase 1 (globoside blood group)	0.84	2.31	0.025
ENSCAFG00845009720	*TXK*	TXK tyrosine kinase	−0.84	2.33	0.044
ENSCAFG00845021928	*TEX14*	testis-expressed 14, intercellular bridge forming factor	0.82	−0.08	0.007
ENSCAFG00845005503	*JAM3*	junctional adhesion molecule 3	−0.81	1.22	0.045
ENSCAFG00845028387	*TOX*	thymocyte selection-associated high-mobility group box	−0.81	2.46	0.021
ENSCAFG00845017349	*GPR84*	G protein-coupled receptor 84	0.80	2.69	0.038
ENSCAFG00845008004	*KLF10*	KLF transcription factor 10	0.79	6.22	0.016
ENSCAFG00845008786	*P2RY2*	purinergic receptor P2Y2	0.78	5.28	0.002
ENSCAFG00845002694	*G0S2*	G0/G1 switch 2	0.78	3.73	0.011
ENSCAFG00845018861 ^1^	(*DSTN*) ^2^	destrin, actin depolymerizing factor	−0.75	1.83	0.000
ENSCAFG00845021223	*AQP3*	aquaporin 3 (Gill blood group)	0.74	4.34	0.013
ENSCAFG00845014621	*SOCS3*	suppressor of cytokine signalling 3	−0.72	3.24	0.038
ENSCAFG00845018321	*ATP10A*	ATPase phospholipid transporting 10A (putative)	−0.72	3.17	0.002
ENSCAFG00845016371	*RGS10*	regulator of G protein signalling 10	−0.70	4.31	0.001
ENSCAFG00845021432	*GNAZ*	G protein subunit α z	−0.70	2.51	0.005
ENSCAFG00845023493	*TSPAN5*	tetraspanin 5	−0.69	3.71	0.047
ENSCAFG00845005802	*VASH1*	vasohibin 1	−0.68	2.33	0.039
ENSCAFG00845019977	*KCNMB4*	potassium calcium-activated channel subfamily M regulatory β subunit 4	−0.68	1.49	0.006
ENSCAFG00845025551 ^1^	(*ATP13A4*) ^2^	ATPase 13A4	−0.66	2.48	0.007
ENSCAFG00845004641	*SPOCK2*	SPARC (osteonectin)-, cwcv-, and kazal-like domains proteoglycan 2	−0.64	6.09	0.025
ENSCAFG00845016402 ^1^	(*GNG11*) ^2^	G protein subunit γ 11	−0.63	5.82	0.010
ENSCAFG00845009033 ^1^	(*CCL14*) ^2^	C-C motif chemokine ligand 14	−0.63	3.34	0.017
ENSCAFG00845000866 ^1^	(*C4H1orf198*) ^2^	chromosome 4 C1orf198 homolog	−0.63	3.29	0.023
ENSCAFG00845008023	*PSEN2*	presenilin 2	−0.62	3.05	0.001
ENSCAFG00845012374	*EHD3*	EH domain containing 3	−0.60	4.48	0.031
ENSCAFG00845025918	*MYL9*	myosin light chain 9	−0.60	5.95	0.028
ENSCAFG00845012329	*APC2*	APC regulator of WNT signalling pathway 2	0.59	1.30	0.011
ENSCAFG00845026987	*ITGB5*	integrin subunit β 5	−0.59	3.04	0.008

^1^ Novel gene; ^2^ UniProtKB Gene Name Symbol.

**Table 3:** Genes modulated in biopsies (T) considering the overall survival (LONG vs. SHORT). Only the DEGs for which a reference gene name was available are listed. Ensembl gene ID, gene name, gene description, log2 fold change (lfc), log2 counts per million (logCPMs), and Benjamini–Hochberg adjusted *p*-value (BHp) are reported.

Ensembl Gene ID	Gene Name	Gene Description	lfc	logCPM	BHp
ENSCAFG00845023887	*KRT76*	keratin 76	8.59	8.08	0.002
ENSCAFG00845014426	*ITGA8*	integrin subunit α 8	5.64	6.34	0.002
ENSCAFG00845000761	*MMP13*	matrix metallopeptidase 13	−4.29	6.51	0.009
ENSCAFG00845010154	*PI16*	peptidase inhibitor 16	8.03	5.88	0.009
ENSCAFG00845004266	*APOA1*	apolipoprotein A1	6.36	3.63	0.009
ENSCAFG00845029417	*MFAP5*	microfibril-associated protein 5	4.52	5.26	0.014
ENSCAFG00845013295	*SVEP1*	sushi, von Willebrand factor type A, EGF, and pentraxin domain containing 1	3.71	5.28	0.019
ENSCAFG00845023760	*ELF5*	E74-like ETS transcription factor 5	4.37	2.07	0.019
ENSCAFG00845028592	*ANKRD55*	ankyrin repeat domain 55	5.82	3.03	0.022
ENSCAFG00845005444	*CDSN*	corneodesmosin	8.82	5.25	0.023
ENSCAFG00845005605	*SOSTDC1*	sclerostin domain containing 1	4.66	3.25	0.023
ENSCAFG00845029513	*WNT5B*	Wnt family member 5B	−2.74	4.04	0.026
ENSCAFG00845001685	*UOX*	urate oxidase	3.98	3.37	0.026
ENSCAFG00845017608	*PLA2G4F*	phospholipase A2 group IVF	3.78	0.59	0.028
ENSCAFG00845025826 ^1^	(*RPTN*) ^2^	repetin	7.33	4.39	0.028
ENSCAFG00845029411	*GDPD2*	glycerophosphodiester phosphodiesterase domain containing 2	4.15	2.21	0.028

^1^ Novel gene; ^2^ UniProtKB Gene Name Symbol.

**Table 4:** Genes modulated in blood samples (B) considering the overall survival (LONG vs. SHORT). Only the DEGs for which a reference gene name was available are listed. Ensembl gene ID, gene name, gene description, log2 fold change (lfc), log2 counts per million (logCPMs), and Benjamini–Hochberg adjusted *p*-value (BHp) are reported.

Ensembl Gene ID	Gene Name	Gene Description	lfc	logCPM	BHp
ENSCAFG00845013314 ^1^	(*LOC111098753*) ^2^		−7.80	−0.68	0.000004
ENSCAFG00845027442	*TKTL1*	Transketolase-like 1	3.04	0.32	0.000004
ENSCAFG00845026120	*H4C4*	H4 clustered histone 4	−7.35	−1.10	0.03

^1^ Novel gene; ^2^ UniProtKB Gene Name Symbol.

**Table 5:** Table summarizing the correlation coefficients (r) and the *p*-values (*p*) of Spearman’s correlation analysis between transcriptional expression level (logCPM) of DEGs in T_OS comparison and OS (days). The *p*-values reported in bold are the significant ones.

Ensembl Gene ID	Gene Name	Gene Description	r	*p*
ENSCAFG00845023887	*KRT76*	keratin 76	0.57	0.15
ENSCAFG00845014426	*ITGA8*	integrin subunit α 8	0.33	0.43
**ENSCAFG00845000761**	***MMP13***	matrix metallopeptidase 13	**−0.79**	**0.03**
ENSCAFG00845010154	*PI16*	peptidase inhibitor 16	0.33	0.43
ENSCAFG00845004266	*APOA1*	apolipoprotein A1	0.52	0.20
ENSCAFG00845029417	*MFAP5*	microfibril-associated protein 5	0.64	0.10
**ENSCAFG00845013295**	***SVEP1***	sushi, von Willebrand factor type A, EGF, and pentraxin domain containing 1	**0.98**	**0.0004**
ENSCAFG00845023760	*ELF5*	E74-like ETS transcription factor 5	0.62	0.12
ENSCAFG00845028592	*ANKRD55*	ankyrin repeat domain 55	0.45	0.27
ENSCAFG00845005444	*CDSN*	corneodesmosin	0.57	0.15
ENSCAFG00845005605	*SOSTDC1*	sclerostin domain containing 1	0.64	0.10
**ENSCAFG00845029513**	***WNT5B***	Wnt family member 5B	**−0.86**	**0.01**
ENSCAFG00845001685	*UOX*	urate oxidase	0.48	0.24
**ENSCAFG00845017608**	***PLA2G4F***	phospholipase A2 group IVF	**0.79**	**0.03**
ENSCAFG00845025826	*RPTN*	repetin	0.36	0.39
ENSCAFG00845029411	*GDPD2*	glycerophosphodiester phosphodiesterase domain containing 2	0.55	0.17
ENSCAFG00845022756	Novel gene ^1^		0.38	0.36
ENSCAFG00845001333	Novel gene ^2^		0.55	0.17

^1^ Uniprot protein name annotation: Ubiquitin–ribosomal protein eS31 fusion protein; ^2^ Uniprot protein name annotation: Elongation factor 1-α.

## Data Availability

Raw Illumina sequencing data have been deposited in GenBank (SRA) under the BioProject accession PRJNA421895.

## Supplementary Materials

The following supporting information can be downloaded at: https://www.mdpi.com/article/10.3390/genes15081065/s1, Table S1: Clinical data of dogs included in this study; Table S2: Histological and immunohistochemical parameters evaluated for each COM enrolled in this study; Table S3: Sequencing and mapping results of blood samples; Table S4: Sequencing and mapping results of tumour samples; Table S5: Differential gene expression analysis results; Table S6: GSEA results; Figure S1: Histopathology of a melanotic and amelanotic melanoma; Figure S2: Immunohistochemistry of Melan-A and Ki-67; Figure S3: Pictures of a patient with oral melanoma before and after radiation and temozolomide therapy; Figure S4: Dotplots representing Spearman’s correlation between *SVEP1*, *MMP13*, *WNT5B*, *PLA2GA7*, and OS.
