# Supplementary material for: A Whole-Transcriptomic Analysis of Canine Oral Melanoma: A Chance to Disclose the Radiotherapy Effect and Outcome-Associated Gene Signature
Source: Genes (Basel). 2024 Aug 13;15(8):1065. doi: 10.3390/genes15081065 (PMC11353338; doi:10.3390/genes15081065)
Supplement: Supplementary file 1 [file genes-15-01065-s001.zip › Mucignat K9 Melanoma_SplOnline.pdf]

genes

MDPI

Supplementary materials

A Whole-Transcriptomic Analysis of Canine Oral Melanoma: A Chance to Disclose the Radiotherapy Effect and Outcome-Associated Gene Signature

Greta Mucignat [1,†], Ludovica Montanucci [2,†], Ramy Elgendy [3], Mery Giantin [1], Paola Laganga [4], Marianna Pauletto [1], Franco Mutinelli [5], Marta Vascellari [5], Vito Ferdinando Leone [4], Mauro Dacasto [1,*] and Anna Granato [5,*]

[1] Department of Comparative Biomedicine and Food Science, University of Padua, Agripolis Legnaro, 35020 Padua, Italy; greta.mucignat@phd.unipd.it (G.M.); mery.giantin@unipd.it (M.G.); marianna.pauletto@unipd.it (M.P.)
[2] McGovern Medical School and Center for Neurogenomics, UTHealth, University of Texas Houston, Houston, TX 77030, USA; ludovica.montanucci@uth.tmc.edu
[3] Discovery Sciences, Centre for Genomics Research, AstraZeneca, 411 10 Gothenburg, Sweden; ramy.elgendy@astrazeneca.com
[4] Anicura—Centro Oncologico Veterinario, Sasso Marconi, 40037 Bologna, Italy; paola.laganga@anicura.it (P.L.); vito.leone@anicura.it (V.F.L.)
[5] Veterinary and Public Health Institute, Legnaro, 35020 Padua, Italy; fmutinelli@izsvenezie.it (F.M.); mvascellari@izsvenezie.it (M.V.)
* Correspondence: mauro.dacasto@unipd.it (M.D.); agranato@izsvenezie.it (A.G.)
† These authors contributed equally to this work.

Table S1. Clinical data of dogs included in the study.

Sample nr.	Breed	Age (yy)/gender	TNM staging	Adjuvant chemotherapy	Outcome
1	Mixed breed	10/M	3 - 0 - 0	TEM	deceased, 132 dd
2	Miniature Schnauzer	15/F	1 - 0 - 0	TEM	deceased, 189 dd
3	Golden Retriever	13/F	3 - 0 - 0	TEM	deceased, 112 dd
4	Mixed breed	14/M	2 - 0 - 0	TEM	deceased, 201 dd
5	Labrador	9/M	3 - 0 - 0	TEM	deceased, 99 dd
6	Miniature Spitz	14/F	3 - 0 - 0	TEM	deceased, 133 dd
7	Golden Retriever	8/M	2 - 0 - 0	TEM	deceased, 270 dd
8	Italian Hound	13/M	3 - 0 - 0	TEM	deceased, 134 dd

dd: days; F: female; M: male; nr.: number; TEM: temozolomide; TNM: primary tumour (T), nodes (N), metastasis (M); yy: years.

Table S2. Histological and immunohistochemical parameters evaluated for each COM enrolled in this study.

Sample nr.	MI	Melanin abundancy	Nuclear atypia grading	MEL-A	PLN-2	Ki-67
1	16	Moderate	5	Y	Y	47.8
2	7	Minimal	4	Y	Y	25.0
3	6	Minimal	6	Y	N	17.8
4	10	Minimal	7	Y	N	30.5
5	11	Minimal	5	Y	Y	32.8
6	5	Moderate	3	Y	N	25.4
7	10	Moderate	3	Y	Y	42.2
8	15	Minimal	6	Y	Y	37

COM: canine oral melanoma; nr.: number; MEL-A: melan-A; MI: mitotic index; N: no; Y: yes.

Table S3. Sequencing and mapping results of blood samples. The table reports the sequenced RNA-seq libraries, including for each of them: i) the number of raw reads obtained; ii) the number of reads after trimming; iii) the number of mapped reads.

Sample	Nr. raw reads	Nr. reads after trimming	Nr. reads mapping
1 T0	32,687,838	32,309,161	27,353,053
1 T1	38,182,037	37,751,300	27,416,567
2 T0	31,241,437	30,857,038	23,245,227
2 T1	31,589,032	31,206,185	23,038,161
3 T0	29,563,457	29,233,024	21,431,650
3 T1	30,975,470	30,524,434	22,536,771
4 T0	39,135,605	38,523,617	28,068,596
4 T1	34,180,603	33,630,994	25,101,733
5 T0	35,026,530	34,575,265	27,426,166
5 T1	42,672,684	42,114,291	34,337,984
6 T0	38,686,328	38,168,355	32,084,760
6 T1	34,714,758	34,157,539	25,951,235
7 T0	45,757,928	45,253,980	34,632,067
7 T1	40,092,730	39,520,675	28,099,707
8 T0	38,372,805	37,923,100	27,040,309
8 T1	44,135,752	43,514,367	30,648,041
**Mean**	36,688,437	36,203,958	27,400,752

Table S4. Sequencing and mapping results of tumour samples. The table reports the sequenced RNA-seq libraries, including for each of them: i) the number of raw reads obtained; ii) the number of reads after trimming; iii) the number of mapped reads.

Sample	Nr. raw reads	Nr. reads after trimming	Nr. reads mapping
1 T0	35,022,278	34,610,558	22,125,257
1 T1	30,255,317	29,839,411	19,972,098
2 T0	25,616,548	25,382,067	17,045,118
2 T1	27,863,695	27,453,097	17,859,291
3 T0	31,881,327	31,424,001	21,779,982
3 T1	41,054,770	40,472,445	25,864,739
4 T0	26,137,877	25,861,050	15,624,906
4 T1	37,320,925	36,845,775	24,137,125
5 T0	33,590,321	33,259,925	23,634,547
5 T1	45,615,378	45,046,701	33,273,957
6 T0	31,961,304	31,592,565	21,672,469
6 T1	34,154,571	33,707,651	22,369,093
7 T0	32,374,540	31,966,654	25,342,377
7 T1	33,752,984	33,281,257	22,509,939
8 T0	34,238,499	33,871,918	19,681,370
8 T1	35,044,189	34,535,414	20,825,935
**Mean**	33,492,783	33,071,906	22,107,388

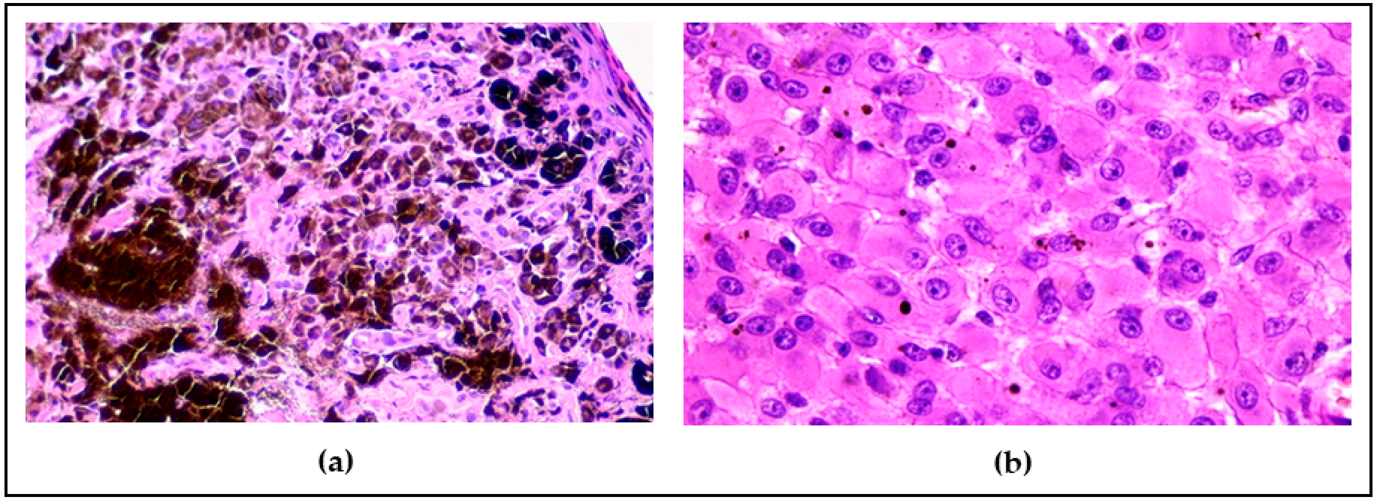

Figure S1. Histopathology of a melanotic **(a)** and amelanotic melanoma **(b)**.

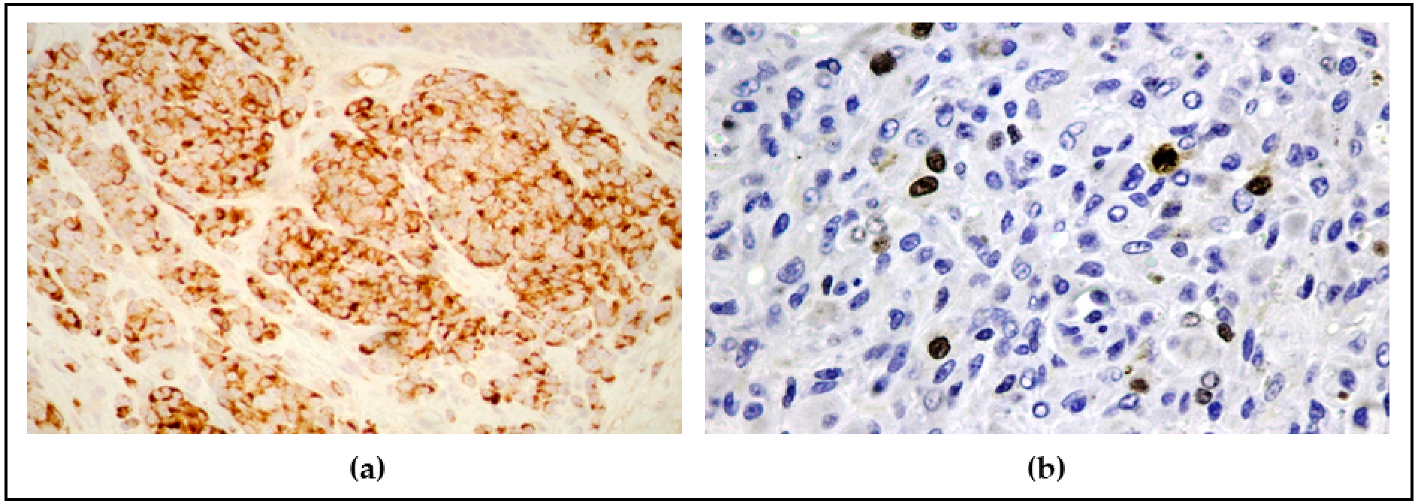

Figure S2. Immunohistochemistry of Melan-A **(a)** and Ki-67 **(b)** positive melanomas.

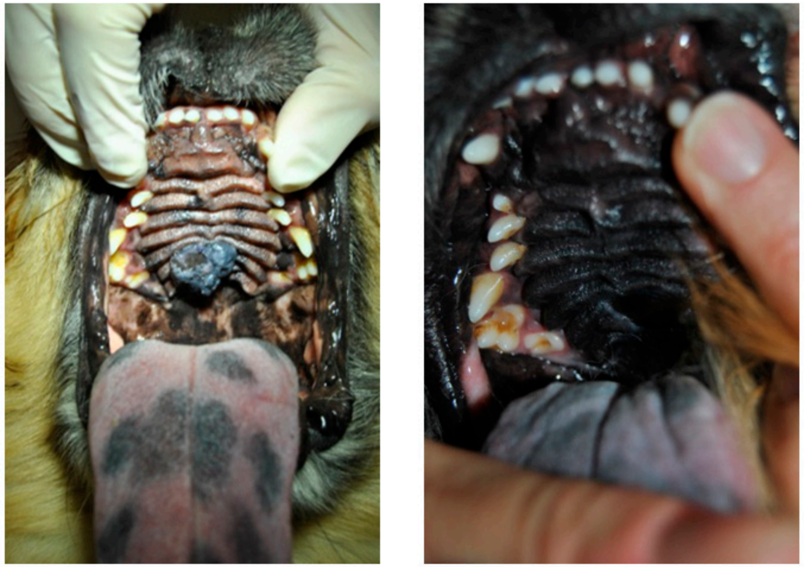

(a) **(b)**

Figure S3. Canine patient with an oral melanoma with a high degree of pigmentation in the palate before **(a)** and after **(b)** radiation therapy plus adjuvant chemotherapy with temozolomide.

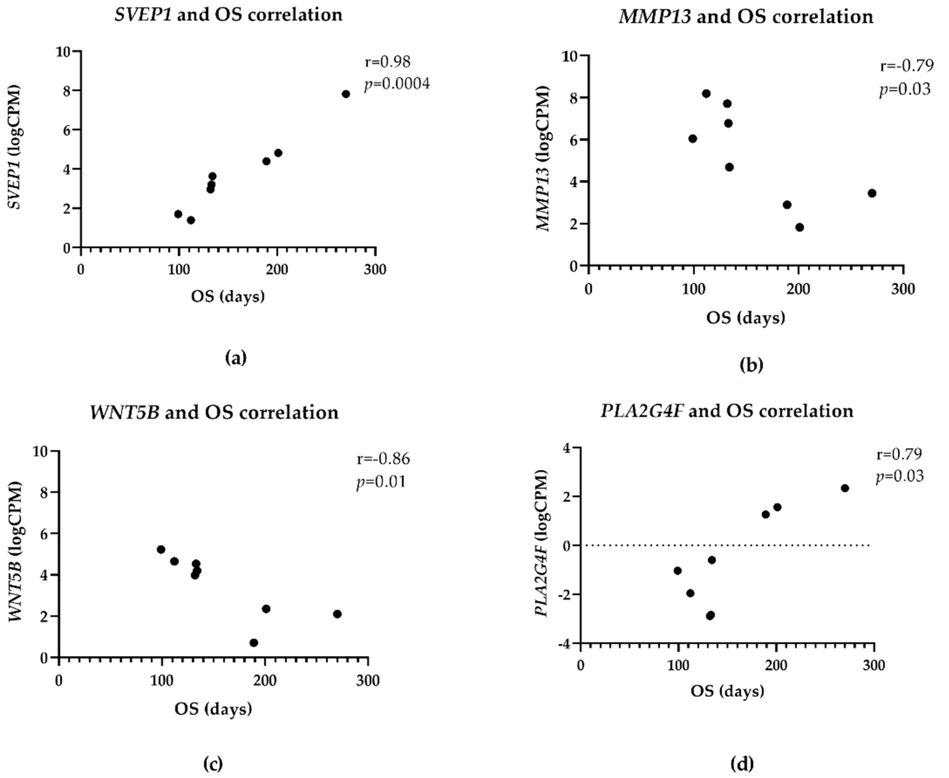

Figure S4. Dotplots representing Spearman's correlation between *SVEP1* **(a)**, *MMP13* **(b)**, *WNT5B* **(c),** and *PLA2G47* **(d)** expression level (logCPM) in tumour samples and OS (days) in the tumour samples of the 8 subjects taken into consideration in the gene expression analysis.
